# New trends in AA amyloidosis with renal involvement: a single-center experience

**DOI:** 10.1590/2175-8239-JBN-2024-0127en

**Published:** 2025-02-03

**Authors:** Nídia Marques, Catarina Oliveira-Silva, Ana Pinho, Ana Teresa Nunes, Susana Sampaio, Ana Oliveira, Isabel Tavares

**Affiliations:** 1 Unidade Local de Saúde São João, Departamento de Nefrologia, Porto, Portugal.; 2 Unidade Local de Saúde de Braga, Departamento de Nefrologia, Braga, Portugal.; 3 Universidade do Porto, Faculdade de Medicina, Porto, Portugal.

**Keywords:** Amyloid Protein AA, Amyloidosis, Epidemiology, Inflammation Mediators, Chronic Infection, Chronic Kidney Disease

## Abstract

**Introduction:**

In the northwest of Portugal, AA amyloidosis was reported as the most frequent amyloid nephropathy, but it is unclear if the disease’s incidence and outcomes have changed. The authors studied the changing epidemiology, aetiologies, and outcomes of patients with renal AA amyloidosis over the last 40 years.

**Methods:**

This is a retrospective single-center cohort study involving patients with renal biopsy-proven AA amyloidosis diagnosed in the northwest of Portugal between 1978 and 2019. The patients were grouped into 14-year cohorts based on the year of diagnosis (CA 1978–1991; CB 1992–2005; CC 2006–2019), and clinical course and outcomes were analyzed.

**Results:**

Sixty-nine AA amyloidosis patients were included. The incidence of the disease remained stable in CA (64%) and CB (62.7%) as opposed to the significant decrease in the most recent cohort (44%, p = 0.027). The mean age at presentation increased by ten years from CA to CC. Overall, infections were the leading cause of death, with a significant rise over time (9.1% in CA to 76.9% at CC, p = 0.002). There were no significant global and renal survival differences between the three cohorts. However, the CC patients died at an older age (61.4 years) than the CA (52.3 years).

**Conclusion:**

The incidence of AA amyloidosis has been declining over the last 40 years. In contrast, the age at presentation of amyloid nephropathy has been increasing. Global and renal outcomes did not improve, but the average life expectancy increased, suggesting progress in general management and supportive care of renal and underlying pathology.

## Introduction

Amyloidosis is a heterogeneous group of rare systemic diseases characterized by the deposition of misfolded proteins in the form of insoluble fibrils^1,2,3^. AA amyloidosis, previously called secondary or reactive amyloidosis, occurs due to deposits of fragments of the acute phase reactant serum amyloid A protein synthesized in the liver^4,5^. Fibril deposition is a well-known consequence of chronic inflammatory and autoinflammatory diseases, cancers, and chronic infections, and it has recently been recognized as an inherited form of AA amyloidosis^2,6,7^. The frequency of AA amyloidosis is decreasing in favor of AL amyloidosis and hereditary amyloidosis, reflecting advances in treating underlying chronic conditions. A recent publication reported a declining frequency of AA amyloidosis from 13% before 2010 to 3% in 2016–2019 in the United Kingdom (UK)^3^. AL amyloidosis is the most prevalent type in Western countries, while AA amyloidosis remains the most common in the developing countries^8^. In our recent Portuguese long-term cohort of patients with renal amyloidosis, AA was the most frequent type (56.1%), followed by AL amyloidosis^9^. This could be attributed to the long study period and the high prevalence of chronic infections as the primary underlying illnesses of AA amyloidosis in Portugal. This contrasts with findings in other developed nations, where underlying diseases vary from infectious to chronic inflammatory conditions^8,9^.

Renal involvement is almost universal in AA amyloidosis, usually presenting as nephrotic syndrome and progressive kidney disease^8^. Renal failure at diagnosis carries a significant morbidity and mortality and, along with hypoalbuminemia, has been suggested as the most important predictor of poor outcomes^10^. The median survival of patients on dialysis treatment ranges from 20 months in American and Turkish reports to 52 months in Spanish studies. However, new data suggest better outcomes for AA amyloidosis over the last two decades^10,11^.

Considering this knowledge, the authors conducted a study to characterize the changing epidemiology, etiologies, and outcomes of patients with renal biopsy-proven AA amyloidosis diagnosed at a major university hospital in the northwest of Portugal in the last 40 years.

## Methods

### Study Design and Patients

We conducted a retrospective cohort study including patients with renal biopsy-proven AA amyloidosis. The study covered the period from January 1978 to December 2019 at Unidade Local de Saúde São João (ULSSJ), a major university hospital in the northwest of Portugal, which serves an estimated population of approximately 2,150,000. The patients were selected from a registry of 3,983 native kidney biopsies. Eligibility criteria included adult patients (>18 years old) with AA amyloid nephropathy and clinical follow-up at our center. Follow-up was defined as the period from kidney disease presentation until the date of death or until August 31, 2020^9^.

Kidney tissue diagnosis and amyloid classification were established using Congo red staining and immunohistochemistry/immunofluorescence with a panel of antibodies targeting serum amyloid A, κ light-chain, λ light-chain, transthyretin, and fibrinogen Aα-chain^9^.

Patients were grouped into 14-year cohorts based on the year of diagnosis (A: 1978–1991, B: 1992–2005, and C: 2006–2019). The selected timeframes resulted from the lack of data in CA and the availability of disease-modifying antirheumatic drugs (DMARD) in CC. Cohort A (CA) included 15 patients, cohort B (CB) 32, and cohort C (CC) 22 patients with AA amyloidosis. Clinical presentation and outcomes (end-stage renal disease (ESRD) and mortality) were analyzed.

All patients’ clinical, laboratory, and demographic data were retrospectively obtained from patient files and electronic hospital records. Demographic and clinical data were recorded, including age at presentation, gender, underlying disease, circumstances of clinical presentation, past medical history, laboratory results such as serum creatinine, serum albumin, serum hemoglobin, and 24h urine protein quantification, extra-renal organ involvement, renal replacement therapy initiation date, and date and cause of death.

The estimated glomerular filtration rate (eGFR) was calculated using the Chronic Kidney Disease Epidemiology Collaboration (CKD-EPI) creatinine equation. Clinical manifestations and stages of chronic kidney disease were classified according to the Kidney Disease Improving Global Outcomes guideline. Definitions of clinically significant extra-renal organ involvement were based on international consensus criteria^9^.

The Health Ethics Committee of the ULSSJ/Faculty of Medicine of the University of Porto approved this study (Deliberation nº 371/2020).

### Statistical Analysis

Categorical variables are presented as absolute values with percentages (%). In contrast, continuous variables are shown as either mean with standard deviation or median with interquartile range [IQR] if they were not normally distributed. Variables with missing data above 25% were marked and not included in the analysis^9^. Cohorts were compared using Pearson’s chi-square (or Fisher’s exact tests correction) for categorical variables and one-way ANOVA or Kruskal-Wallis test for continuous variables, as appropriate.

Global survival (time from kidney disease diagnosis to death), renal survival (time from kidney disease diagnosis to ESRD, censored for death), and survival on dialysis (time from dialysis commencement to death, censored for kidney transplantation) were estimated by Kaplan-Meier analysis and Poisson regression^9^. Differences between curves were evaluated using the log-rank test. A p < 0.05 was considered significant. All statistical analyses were performed using the IBM SPSS version 26.0 software.

## Results

### Characterization of Patients

Sixty-nine patients diagnosed with AA amyloidosis over the last four decades were included. This corresponded to 56.1% of all amyloid nephropathy biopsies from the same period and 1.7% of all nephropathologic diagnoses performed at our center^9^. The incidence of AA amyloidosis was 1 case per million person-years.

Demographic and clinical characteristics are listed in Table 1. Forty-nine percent of patients were male, and almost all were white. The mean age at kidney disease presentation was 48.6 ± 15.4 years. Patients with underlying chronic infectious diseases were older than those with chronic inflammatory conditions (53.1 vs. 44.9 years, respectively). More than half of the patients were at least in CKD stage 3 at presentation and presented nephrotic range proteinuria. Chronic infections were the leading cause of AA amyloidosis, corresponding to 47.8% of patients (Table 2).

**Table 1:** Clinical characteristics of patients with AA amyloidosis

	CA (1978–1991) (n = 15)	CB (1991–2005) (n = 32)	CC (2006–2019) (n = 22)	Overall (n = 69)	p
**Demography**					
Gender Male	8 (53.3)	15 (46.9)	11 (50)	34 (49.3)	0.915
Ethnic group (white)	14 (100)	32 (100)	21 (95.5)	68 (98.6)	0.338
**Kidney disease presentation**					
Age at presentation, y	40.9 ± 17.8	50.8 ± 15.3	50.5 ± 12.8	48.6 ± 15.4	0.095
Chronic infectious diseases	50.1 ± 14.5	58.3 ± 12.9	48.8 ± 14.4	53.1 ± 14.1	0.191
Chronic inflammatory diseases	25.8 ± 9.8	44.5 ± 15.0	54.5 ± 11.5	44.9 ± 15.8	**0.002**
Duration of underlying disease at presentation, mo	152.0 [50.2–252.5]	130.3 [93.8–246.2]	57.4 [11.9–215.4]	115.0 [38.8–232.7]	0.205
Proteinuria, g/24h					0.829
< 1	1 (7.1)	3 (9.7)	4 (19.0)	8 (12.1)	
1–3	5 (35.7)	10 (32.3)	6 (28.6)	21 (31.8)	
> 3	8 (58.1)	18 (58.1)	11 (52.4)	37 (56.0)	
eGFR categories, mL/min/1.73 m^2^					0.527
< 30	3 (20.0)	7 (21.9)	7 (31.8)	17 (24.6)	
30–60	3 (20.0)	11 (34.4)	8 (36.4)	22 (31.9)	
> 60	9 (60.0)	14 (63.6)	7 (31.8)	30 (43.5)	
eGFR absolute value, mL/min/1.73 m^2^	82.1 ± 48.3	63.2 ± 38.7	49.9 ± 36.9	63.1 ± 41.4	0.067
**At renal biopsy**					
Proteinuria, g/24h	5.5 [3.1–6.5]	4.3 [2.1–9.6]	5.8 [4.2–6.7]	5.5 [2.5–7.6]	0.636
eGFR, mL/min/1.73 m^2^					0.051
< 30	4 (26.7)	8 (25.0)	11 (50)	23 (33.3)	
30–60	1 (6.7)	10 (31.3)	6 (27.3)	17 (24.6)	
> 60	10 (66.7)	14 (43.7)	5 (22.7)	29 (42.0)	
eGFR absolute value, mL/min/1.73 m^2^	106.9 [18.8 – 132.1]	50.9 [30.5 – 98.3]	33.6 [8.9 – 60.6]	54.1 [30.0 – 98.9]	**0.020**
Serum albumin, g/dL	1.5 ± 0.7	2.2 ± 0.9	2.6 ± 0.9	2.2 ± 0,9	**0.001**
Hemoglobin, g/dL	12.0 ± 2.9	12.1 ± 1.8	10.6 ± 2.4	11.6 ± 2.3	**0.038**
HTN	3 (23.1)	13 (38.2)	14 (63.6)	30 (43.5)	**0.029**

Abbreviations – y: years; mo: months; wk: weeks; eGFR: estimated glomerular filtration rate; wk: weeks; HTN: arterial hypertension. Notes – Values for categorical variables are given as numbers (percentage); values for continuous variables as mean ± standard deviation if normally distributed or median [interquartile range] if non-normally distributed. p-Values are from Pearson’s chi-square (or Fisher’s exact tests correction) for categorical variables and one-way ANOVA or Kruskal-Wallis test for continuous variables, as appropriate.

**Table 2:** Underlying diseases of patients with AA amyloidosis

	CA (1978–1991) (n = 15)	CB (1992–2005) (n = 32)	CC (2006–2019) (n = 22)	Overall (n = 69)	p
**Underlying disease**					
**Chronic infection**	**7 (46.7)**	**14 (43.8)**	**12 (54.5)**	**33 (47.8)**	0.734
Tuberculosis	4	6	4	14 (20.3)	
Bronchiectasis	1	4	3	8 (11.6)	
Injection drug abuse	0	2	1	3 (4.3)	
Cystic fibrosis	0	0	2	2 (2.9)	
Chronic ulcers	2	0	0	2 (2.9)	
Osteomyelitis	0	0	1	1 (1.4)	
Whipple disease	0	0	1	1 (1.4)	
Recurrent pulmonary infections	0	1	0	1 (1.4)	
Xanthogranulomatous pyelonephritis	0	1	0	1 (1.4)	
**Chronic inflammatory diseases**	**4 (26.7)**	**17 (53.1)**	**7 (31.8)**	**28 (40.6)**	0.136
**Rheumatic diseases**	**4 (26.7)**	**13 (40.6)**	**4 (18.2)**	**21 (30.4)**	0.199
Rheumatoid arthritis	0	9	2	11 (15.9)	
Juvenile idiopathic arthritis	3	1	0	4 (5.8)	
Psoriatic arthritis	0	1	1	2 (2.9)	
Ankylosing spondylitis	0	2	0	2 (2.9)	
Systemic lupus erythematosus	1	0	0	1 (1.4)	
**Other chronic inflammatory diseases**	**0 (0)**	**4 (12.5)**	**3 (13.6)**	**7 (10.1)**	0.336
Crohn’s disease	0	2	2	4 (5.8)	
Polymyalgia rheumatica	0	0	1	1 (1.4)	
Hidradenitis suppurativa	0	0	0	1 (1.4)	
Panniculitis associated with α1-antitrypsin deficiency	0	1	0	1 (1.4)	
Muckle-Wells syndrome (*NLRP3* p.Arg262Leu)	0	1	0	1 (1.4)	
**Malignant diseases**	**2 (13.3)**	**0 (0)**	**2 (9.1)**	**4 (5.8)**	0.138
Colorectal carcinoma	1	0	0	1 (1.4)	
Cervical cancer	0	0	1	1 (1.4)	
Breast cancer	0	0	1	1 (1.4)	
Non-Hodgkin’s lymphoma	1	0	0	1 (1.4)	
**Idiopathic**	**2 (13.3)**	**1 (2.9)**	**1 (4.5)**	**4 (5.8)**	0.360

Notes – Values for categorical variables are given as numbers (percentages).

The median duration of follow-up was 37.7 months (Table 3). About 74% of patients progressed to ESRD, and 13% had ESRD at presentation (Table 3). Only two patients underwent kidney transplantation.

**Table 3:** Clinical course and outcomes of AA amyloidosis

	1978–1991 (n = 15)	1992–2005 (n = 32)	2006–2019 (n = 22)	Overall (n = 69)	p
**Follow-up**, mo^a^	12.8 [4.0–229.2]	60.4 [8.3–151.1]	31.7 [11.2–74.7]	37.7 [8.2–134.7]	0.666
**Extra-renal involvement** ^b^	4 (26.7)	9 (28.1)	14 (63.6)	27 (39.1)	**0.017**
One organ involvement	3 (20.0)	4 (12.5)	11 (50)	18 (26.1)	**0.008**
Multiorgan involvement	1 (6.7)	5 (15.6)	3 (13.6)	9 (13)	0.693
Cardiac	0 (0)	6 (18.8)	5 (22.7)	11 (15.9)	0.286
Hepatic	1 (6.7)	3 (9.4)	4 (18.2)	8 (11.6)	0.707
Neuropathy	2 (13.3)	0 (0)	1 (4.5)	3 (4.3)	**0.006**
Orthostatic hypotension	0 (0)	3 (9.4)	1 (4.5)	4 (5.8)	0.420
Gastro-intestinal tract	1 (6.7)	3 (9.4)	6 (27.3)	10 (14.5)	0.145
Thyroid	–	3 (9.4)	1 (4.5)	4 (5.8)	NA^c^
**Clinical outcomes**					
**Renal replacement therapy**					
ESRD requiring dialysis	10 (66.7)	23 (71.9)	18 (81.8)	51 (73.9)	0.551
ESRD at presentation	1 (6.7)	3 (9.4)	5 (22.7)	9 (13.0)	0.255
ESRD during follow-up	9 (60.0)	20 (62.5)	13 (59.1)	42 (60.9)	0.751
Kidney transplantation	1 (6.7)	1 (3.1)	0 (0)	2 (2.9)	0.157
**Death**	13 (86.7)	29 (90.6)	13 (59.1)	55 (79.7)	**0.014**
Infection^d^	1 (9.1)	15 (48.4)	10 (76.9)	26 (47.3)	**0.002**
Heart failure	1 (9.1)	4 (12.9)	0 (0)	4 (7.3)	
Isquemic events	0 (0)	5 (16.1)	1 (7.7)	7 (10.1)	
Sudden death or cardiac arrhythmia	2 (18.2)	0 (0)	1 (7.7)	3 (5.5)	
Renal failure	1 (9.1)	1 (3.2)	0 (0)	2 (3.6)	
Miscellaneouse	3 (27.3)	1 (3.2)	0 (0)	4 (7.3)	
Deaths unrelated to amyloidosis	0 (0)	1 (3.2)	1 (7.7)	2 (3.6)	
Unknown	5 (38.5)	2 (6.9)	0 (0)	7 (12.7)	

Abbreviations – mo: months; end-stage renal disease: ESRD. Notes – Values for categorical variables are given as numbers (percentage) and continuous variables as median [interquartile range]. p-Values are from Pearson’s chi-square (or Fisher’s exact tests correction) for categorical variables and Kruskal-Wallis test for continuous variables, as appropriate. ^a^End of follow-up: August 2020. ^b^Missing data above 25% (percentages relate to the total number of patients). ^c^Not applicable for this category. ^d^Infection included sepsis (n = 20), pneumonia (n = 4), tuberculous empyema (n = 1), peritonitis (n = 1). eMiscellaneous causes included pulmonary hemorrhage (n = 1), colorectal cancer (n = 2), amyloidosis (n = 1).

As shown in Table 4, the median time to ESRD was 31 months. Median global survival was 66 [95% CI 33.0–99.0] months, and the life expectancy of these patients was 64 [95% CI 56.2–71.8] years. Infections were the leading cause of death (Table 3).

**Table 4:** Survival data of patients with AA amyloidosis

	CA (1978–1991) (n = 15)	CB (1992–2005) (n = 32)	CC (2006–2019) (n = 22)	Overall (n = 69)	p
**Global survival, mo** ^a^	14 [95% CI 0.0–131.4]	66 [95% CI 48.0–84.0]	42 [95% CI 0.0–104.2]	66 [95% CI 33.0–99.0]	0.784
**Renal survival, mo** ^a^	103 [95% CI 14.5–191.5]	22 [95% CI 0.5–43.5]	26 [95% CI 8.8–43.2]	31 [95% CI 14.6–47.3]	0.504
**Survival on dialysis, mo** ^b^	8 [95% CI 0.0–18.8]	21 [95% CI 0.5–41.3]	19 [95% CI 8.6–29.4]	20 [95% CI 12.0–28.0]	0.969
**Age at death, y** ^c^	52.3 [95% CI 43.7–60.8]	59.0 [95% CI 53.6–64.4]	61.4 [95% CI 54.2–68.5]	58.3 [95% CI 54.5–62.2]	0.217
Chronic infection	58.3 [95% CI 47.7–68.9]	62.4 [95% CI 55.1–69.6]	56.4 [95% CI 46.8–66.0]	59.4 [95% CI 54.1–64.6]	0.727
Chronic inflammatory diseases	44.5 [95% CI 29.7–59.3]	55.7 [95% CI 47.6–63.8]	71.2 [95% CI 62.2–80.2]	57.5 [95% CI 51.1–63.9]	**0.041**
**1-year death rate/100 patient-year**	8.97	12.5	11.4	11.2	
**1-year ESRD rate/100 patient-year**	10.98	17.4	24.7	17.2	

Abbreviations – y: years; mo: months. Notes – ^a^Values are given as medium. ^b^Survival on dialysis of 51 patients with AA amyloidosis, censored at renal transplantation. ^c^Values are given as mean.

### Comparison of Cohorts

**Figure 1:**
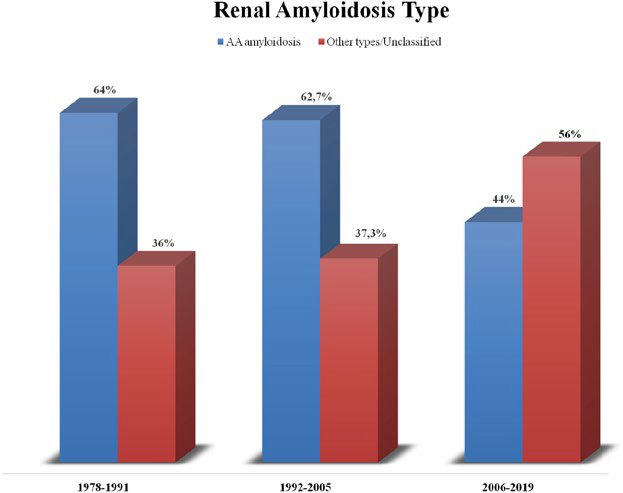
Incidence of AA amyloidosis in the last 40 years.

The incidence of AA amyloidosis remained stable in CA and CB as opposed to the significant decrease in the most recent cohort (64%, 62.7%, and 44%, respectively, *p =* 0.027) as shown in Figure 1.

### Baseline Characteristics and Underlying Diseases

As shown in Table 1, gender distribution remained stable across the three cohorts, with men and women being equally affected. The mean age at presentation increased from 40.9 years in CA to 50.5 in CC (p = 0.095). When analyzing patients by the underlying cause, those with chronic inflammatory conditions had the most significant increase in the mean age at kidney disease presentation, increasing from 25.8 in CA to 54.5 in CC (p = 0.002).

Median eGFR at biopsy decreased along the three cohorts, from 106.9 mL/min/1.73 m^2^ to 33.6 mL/min/1.73 m^2^ (p = 0.020), and more patients were in CKD stage 4 or higher at the time of kidney biopsy in CC (50%) than in CA (26.7%) (p = 0.051). Serum albumin and hemoglobin also showed significant differences over the three cohorts. Serum albumin improved from a mean of 1.5 g/dL in CA to 2.6 g/dL in CC (p = 0.001), but hemoglobin decreased from 12.0 g/dL to 10.6 g/dL (p = 0.038). Hypertension was significantly more common in CC (63.6%) than in CA (23.1%) (p = 0.029).

There were no changes in the relative percentages of the underlying diseases across the three cohorts (Table 2). Chronic infections were the primary underlying disease, slightly increasing over time (from 46.7% in CA to 54.5% in CC), although not statistically significant. The incidence of tuberculosis decreased from CA to CB, noting that no cases were due to this infection since 2009.

### Clinical Course and Outcomes

All patients completed the study, and there were no losses to follow-up. Extra-renal involvement was more frequent in CC (63.6%) than in CA (23.1%) (p = 0.017) (Table 4). Overall, infections were the leading cause of death, with a significant rise over time (9.1% in CA to 76.9% at CC, p = 0.002).

There were no statistically significant global and renal survival changes across the three cohorts (Table 4). Median renal survival decreased from 103 to 26 months, according to the increase in the 1-year ESRD rate/100 patient-year. Median global survival increased from 14 to 42 months, and the 1-year death rate/100 patient-year remained stable, which was not statistically significant even considering cardiac involvement. CC patients died at an older age (61.4 years) than CA patients (52.3 years) (p = 0.217). Considering etiology, the average life expectancy improved in patients with underlying chronic inflammatory conditions from 44.5 years in CA to 71.2 years in CC (p = 0.041).

## Discussion

In the last four decades, AA amyloidosis was the most frequent type of amyloid nephropathy in our center. Our biopsy incidence of renal AA amyloidosis was 56.1%, which significantly differs from the 7% reported by the Mayo Clinic^12^, and from the 12% reported by the UK National Amyloidosis Centre (NAC), although the last series included tissue biopsies other than kidney^13^. We observed a decline in cases of AA amyloidosis from 62.7% before 2006 to 44% after 2006. This aligns with contemporary trends reported by the UK NAC database, although it remains exceptionally high^3^. Nonetheless, our incidence rate of AA amyloidosis is about 1 case per million person-years, which is in line with the reported incidence of the disease in Western countries^14^.

The most recent series from UK NAC reported a decline in AA amyloidosis cases to 3% after 2016 and an increase in cases of ATTRwt amyloidosis^3^. In our series, the decrease in tuberculosis incidence and the increase in cases of AL amyloidosis were the leading causes of the epidemiologic change in recent years^9^. However, AL amyloidosis remains underdiagnosed considering the minimum estimated incidence of 0.5 cases per 100,000 and our reference area greater than 1 million inhabitants^15^. The results of a recent Delphi panel applied to Portuguese physicians from different medical specialties revealed a lack of consensus regarding the diagnosis and early management of patients with AL amyloidosis, which may contribute to its underdiagnosis^16^. Another explanation for the higher incidence of the AA type is the inclusion of patients with kidney biopsy-proven amyloid, which could overestimate the relative percentage of predominant renal amyloidosis as AA amyloidosis. In general, we do not have an excess of AA, but rather a deficit in the diagnosis of other forms of amyloidosis with renal involvement, namely AL.

In our series, patients were significantly older at diagnosis in the most recent cohort, with a mean age of 50.5 years. This probably reflects an improvement in treating underlying conditions, particularly inflammatory diseases, and it is in line with other reports, although our patients were younger^11,14,17^.

Differently than other developed countries, where inflammatory conditions predominate, in our cohort, infectious diseases remained the leading cause of AA amyloidosis across the three cohorts^11,18^. Overall, tuberculosis was the most common infection, which is related to the lengthened study period and the high tuberculosis incidence rate in Portugal. The high frequency of associated AA amyloidosis may have been influenced by post-tuberculosis bronchiectasis, primarily seen in the oldest cohorts. In the past, tuberculosis was documented in many series as the main cause of AA amyloidosis, but the incidence has decreased recently^5^. At our facility, there have been no confirmed cases of tuberculosis-related AA amyloidosis since 2009.

Although tuberculosis cases decreased, new infections like bronchiectasis and chronic osteomyelitis became more prevalent. Due to increasing age and comorbidities, source control is not always possible, and chronic inflammation persists. Conversely, there was a trend for reduction of chronic rheumatological conditions, such as rheumatoid arthritis, as causes of AA form, probably attributable to the availability of DMARD. In contrast to growing evidence, intravenous drug abuse was not found to be associated with AA amyloidosis in our cohort^11^.

Patients most recently diagnosed were older and presented with lower renal function at kidney biopsy. This could be related to a delay in patient referral to nephrology due to older age at presentation, comorbidities, and low suspicion for amyloidosis. Renal functional reserve decreases with age, which could lead to a more rapid loss of renal function in the presence of this pathology^11^. The low hemoglobin and the prevalence of hypertension in cohort CC at presentation were probably related to more advanced kidney failure.

In line with the older age at diagnosis, the age at death increased from 52.3 to 61.4 years old. This was most evident when the underlying etiology was inflammatory conditions, reflecting better disease control and later complications, considering the emergence of new therapies. However, global survival did not differ significantly. This was probably related to more advanced age and worse kidney disease at presentation in the last decade. The same explanations justify our renal survival findings.

Infectious diseases were the leading cause of death of these patients in the last thirty years, with an increasing incidence. The availability of DMARDs dramatically changed the natural course of rheumatologic conditions. The cumulative immunosuppressive effect of DMARDs combined with an increase in life expectancy and age frailty could be one explanation for this trend.

We acknowledge certain limitations to this study conducted outside a referral center for hereditary amyloidosis^9^. The study’s long period and retrospective design are susceptible to information bias, and the limited available data, particularly from diagnoses established in earlier decades, has restricted the analysis of some variables. Furthermore, patient selection based on renal biopsy restricted the sample size. However, the study’s main strength lies in its long-term nature, with follow-up periods exceeding those of some of the most comprehensive studies on systemic amyloidosis survival^9^.

In conclusion, the results of our retrospective cohort show a decreasing incidence of AA amyloidosis in the northwest of Portugal. Life expectancy improved overall, reflecting advances in therapeutics and management of these patients. Nonetheless, renal prognosis remained unaltered over the decades, suggesting the need for adequate recognition, earlier diagnosis, and timely nephrology referral, as the disease appears later in life.
