# Supplementary material for: New trends in AA amyloidosis with renal involvement: a single-center experience
Source: J Bras Nefrol. 2025 Feb 3;47(2):e20240127. doi: 10.1590/2175-8239-JBN-2024-0127en (PMC11801712; doi:10.1590/2175-8239-JBN-2024-0127en)
Supplement: Translation

# Translation: Novas tendências na amiloidose AA com envolvimento renal: experiência de um único centro

**DOI:** 10.1590/2175-8239-JBN-2024-0127pt

**Published:** 2025-01-25

**Authors:** Nídia Marques, Catarina Oliveira-Silva, Ana Pinho, Ana Teresa Nunes, Susana Sampaio, Ana Oliveira, Isabel Tavares

**Affiliations:** 1 Unidade Local de Saúde São João, Departamento de Nefrologia, Porto, Portugal.; 2 Unidade Local de Saúde de Braga, Departamento de Nefrologia, Braga, Portugal.; 3 Universidade do Porto, Faculdade de Medicina, Porto, Portugal.

**Keywords:** Proteína Amiloide A Sérica, Amiloidose, Epidemiologia, Mediadores da Inflamação, Infecção Crônica, Doença Renal Crônica.

## Abstract

**Introdução:**

No noroeste de Portugal, a amiloidose AA foi relatada como a nefropatia amiloide mais frequente, embora não esteja claro se a incidência e desfechos da doença mudaram. Os autores analisaram alterações na epidemiologia, etiologias e desfechos de pacientes com amiloidose renal AA nos últimos 40 anos.

**Métodos:**

Estudo de coorte retrospectivo de centro único, envolvendo pacientes com diagnóstico de amiloidose AA comprovada por biópsia renal, realizado no noroeste de Portugal entre 1978 e 2019. Os pacientes foram agrupados em coortes de 14 anos baseadas no ano do diagnóstico (CA 1978–1991; CB 1992–2005; CC 2006–2019), com análises de evolução clínica e desfechos.

**Resultados:**

Incluímos 69 pacientes com amiloidose AA. A incidência da doença permaneceu estável em CA (64%) e CB (62,7%), em oposição à redução significativa na coorte mais recente (44%, p = 0,027). A idade média na apresentação aumentou em dez anos de CA para CC. No geral, infecções foram a principal causa de óbito, com aumento significativo ao longo do tempo (9,1% na CA para 76,9% na CC; p = 0,002). Não houve diferenças significativas de sobrevida global e renal entre as três coortes. Entretanto, pacientes na CC faleceram com idade mais avançada (61,4 anos) do que na CA (52,3 anos).

**Conclusão:**

A incidência de amiloidose AA tem diminuído nos últimos 40 anos. Em contrapartida, a idade de apresentação da nefropatia amiloide tem aumentado. Desfechos globais e renais não melhoraram, mas a expectativa de vida média aumentou, sugerindo progresso no manejo geral e no tratamento de suporte da patologia renal e subjacente.

## Introdução

A amiloidose é um grupo heterogêneo de doenças sistêmicas raras caracterizadas pela deposição de proteínas mal dobradas na forma de fibrilas insolúveis^1,2,3^. A amiloidose AA, anteriormente denominada amiloidose secundária ou reativa, ocorre devido a depósitos de fragmentos da proteína amiloide A sérica, reagente de fase aguda, sintetizada no fígado^4,5^. A deposição de fibrilas é uma consequência amplamente conhecida de doenças inflamatórias e autoinflamatórias crônicas, cânceres e infecções crônicas, tendo sido recentemente reconhecida como uma forma hereditária de amiloidose AA^2,6,7^. A frequência da amiloidose AA está diminuindo em favor da amiloidose AL e da amiloidose hereditária, refletindo os avanços no tratamento de doenças crônicas subjacentes. Uma publicação recente relatou uma redução na frequência de amiloidose AA no Reino Unido (GB), de 13% antes de 2010 para 3% entre 2016 e 2019^3^. A amiloidose AL é o tipo mais prevalente nos países ocidentais, enquanto a amiloidose AA continua sendo a mais comum nos países em desenvolvimento^8^. Em nossa recente coorte de longo prazo de pacientes com amiloidose renal em Portugal, a amiloidose AA foi o tipo mais frequente (56,1%), seguida pela amiloidose AL^9^. Isso pode ser atribuído ao longo período de estudo e à alta prevalência de infecções crônicas como as principais doenças subjacentes à amiloidose AA no país. Esse dado contrasta com os achados em outros países desenvolvidos, onde as doenças subjacentes variam de condições infecciosas a inflamatórias crônicas^8,9^.

O envolvimento renal é quase universal na amiloidose AA, geralmente manifestando-se como síndrome nefrótica e doença renal progressiva^8^. A insuficiência renal no momento do diagnóstico acarreta morbidade e mortalidade significativas e, juntamente com a hipoalbuminemia, tem sido sugerida como o preditor mais importante de desfechos desfavoráveis^10^. A mediana de sobrevida de pacientes em tratamento dialítico varia de 20 meses, segundo relatos americanos e turcos, a 52 meses, segundo estudos espanhóis. Entretanto, novos dados sugerem melhores desfechos para a amiloidose AA nas últimas duas décadas^10,11^.

Considerando tal conhecimento, os autores realizaram um estudo para caracterizar as alterações na epidemiologia, etiologias e desfechos de pacientes com amiloidose AA comprovada por biópsia renal, diagnosticados em um importante hospital universitário no noroeste de Portugal nos últimos 40 anos.

## Métodos

### Desenho do estudo e pacientes

Realizamos um estudo de coorte retrospectivo, incluindo pacientes com amiloidose AA comprovada por biópsia renal. O estudo abrangeu o período de janeiro de 1978 a dezembro de 2019 na Unidade Local de Saúde São João (ULSSJ), um importante hospital universitário no noroeste de Portugal, que atende a uma população estimada de aproximadamente 2.150.000 habitantes. Os pacientes foram selecionados a partir de um registro de 3.983 biópsias em rins nativos. Os critérios de elegibilidade incluíram pacientes adultos (>18 anos de idade) com nefropatia amiloide AA e acompanhamento clínico em nosso centro. O acompanhamento foi definido como o período desde a apresentação da doença renal até a data do óbito ou até 31 de agosto de 2020^9^.

O diagnóstico do tecido renal e a classificação da amiloidose foram estabelecidos com o uso de coloração com vermelho Congo e imuno-histoquímica/imunofluorescência utilizando um painel de anticorpos direcionados à proteína amiloide A sérica, cadeia leve κ, cadeia leve λ, transtirretina e cadeia Aα do fibrinogênio^9^.

Os pacientes foram agrupados em coortes de 14 anos com base no ano de diagnóstico (A: 1978–1991, B: 1992–2005 e C: 2006–2019). Os períodos de tempo selecionados resultaram da falta de dados na CA e da disponibilidade de medicamentos antirreumáticos modificadores da doença (DMARD, por sua sigla em inglês) na CC. A coorte A (CA) incluiu 15 pacientes, a coorte B (CB) 32 e a coorte C (CC) 22 pacientes com amiloidose AA. Foram analisados a apresentação clínica e os desfechos (doença renal em estágio terminal [DRET] e mortalidade).

Todos os dados clínicos, laboratoriais e demográficos dos indivíduos foram obtidos retrospectivamente a partir dos prontuários dos pacientes e registros eletrônicos do hospital. Foram registrados dados demográficos e clínicos, incluindo idade na apresentação, sexo, doença subjacente, circunstâncias da apresentação clínica, histórico médico, resultados laboratoriais como creatinina sérica, albumina sérica, hemoglobina sérica e quantificação de proteína na urina de 24 horas, envolvimento de órgãos extrarrenais, data de início da terapia renal substitutiva e data e causa do óbito.

A taxa de filtração glomerular estimada (TFGe) foi calculada utilizando a equação de creatinina CKD-EPI (*Chronic Kidney Disease Epidemiology Collaboration*). As manifestações clínicas e os estágios da doença renal crônica foram classificados de acordo com as diretrizes do KDIGO (*Kidney Disease Improving Global Outcomes*). As definições de envolvimento clinicamente significativo de órgãos extrarrenais foram baseadas em critérios de consenso internacional^9^.

O Comitê de Ética em Saúde da ULSSJ/Faculdade de Medicina da Universidade do Porto aprovou este estudo (Deliberação nº 371/2020).

### Análise Estatística

As variáveis categóricas são apresentadas como valores absolutos com porcentagens (%). Em contrapartida, as variáveis contínuas são mostradas como média com desvio padrão ou mediana com intervalo interquartil [IIQ] caso não apresentem distribuição normal. Variáveis com dados ausentes superiores a 25% foram sinalizadas e não incluídas na análise^9^. As coortes foram comparadas por meio do teste qui-quadrado de Pearson (ou correção pelo teste exato de Fisher) para variáveis categóricas e ANOVA unidirecional ou teste de Kruskal-Wallis para variáveis contínuas, conforme apropriado.

A sobrevida global (tempo desde o diagnóstico da doença renal até o óbito), a sobrevida renal (tempo desde o diagnóstico da doença renal até a DRET, censurada para óbito) e a sobrevida em diálise (tempo desde o início da diálise até o óbito, censurada para transplante renal) foram estimadas pela análise de Kaplan-Meier e regressão de Poisson^9^. As diferenças entre as curvas foram avaliadas com o teste log-rank. Um valor de p < 0,05 foi considerado significativo. Todas as análises estatísticas foram realizadas utilizando o software IBM SPSS versão 26.0.

## Resultados

### Caracterização de Pacientes

Foram incluídos 69 pacientes diagnosticados com amiloidose AA ao longo das últimas quatro décadas. Isso correspondeu a 56,1% de todas as biópsias de nefropatia amiloide do mesmo período e a 1,7% de todos os diagnósticos nefropatológicos realizados em nosso centro^9^. A incidência de amiloidose AA foi de 1 caso por milhão de pessoas-ano.

As características demográficas e clínicas estão listadas na Tabela 1. Quarenta e nove por cento dos pacientes eram do sexo masculino e quase todos eram brancos. A média de idade na apresentação da doença renal foi de 48,6 ± 15,4 anos. Os pacientes com doenças infecciosas crônicas subjacentes apresentaram idade mais avançada do que aqueles com doenças inflamatórias crônicas (53,1 *vs.* 44,9 anos, respectivamente). Mais da metade dos pacientes encontrava-se pelo menos no estágio 3 da DRC no momento da apresentação, além de apresentar proteinúria na faixa nefrótica. As infecções crônicas foram a principal causa de amiloidose AA, correspondendo a 47,8% dos pacientes (Tabela 2).

**Tabela 1:** Características clínicas dos pacientes com amiloidose AA

	CA (1978–1991) (n = 15)	CB (1991–2005) (n = 32)	CC (2006–2019) (n = 22)	Total (n = 69)	p
**Demografia**					
Sexo					
Masculino	8 (53,3)	15 (46,9)	11 (50)	34 (49,3)	0,915
Grupo étnico (branco)	14 (100)	32 (100)	21 (95,5)	68 (98,6)	0,338
**Apresentação da doença renal**					
Idade na apresentação, a	40,9 ± 17,8	50,8 ± 15,3	50,5 ± 12,8	48,6 ± 15,4	0,095
Doenças infecciosas crônicas	50,1 ± 14,5	58,3 ± 12,9	48,8 ± 14,4	53,1 ± 14,1	0,191
Doenças inflamatórias crônicas	25,8 ± 9,8	44,5 ± 15,0	54,5 ± 11,5	44,9 ± 15,8	**0,002**
Duração da doença subjacente na apresentação, me	152,0 [50,2–252,5]	130,3 [93,8–246,2]	57,4 [11,9–215,4]	115,0 [38,8 –232,7]	0,205
Proteinúria, g/24h					0,829
< 1	1 (7,1)	3 (9,7)	4 (19,0)	8 (12,1)	
1–3	5 (35,7)	10 (32,3)	6 (28,6)	21 (31,8)	
> 3	8 (58,1)	18 (58,1)	11 (52,4)	37 (56,0)	
Categorias da TFGe, mL/min/1,73 m^2^					0,527
< 30	3 (20,0)	7 (21,9)	7 (31,8)	17 (24,6)	
30–60	3 (20,0)	11 (34,4)	8 (36,4)	22 (31.9)	
> 60	9 (60,0)	14 (63,6)	7 (31,8)	30 (43,5)	
Valor absoluto da TFGe, mL/min/1,73 m^2^	82,1 ± 48,3	63,2 ± 38,7	49,9 ± 36,9	63,1 ± 41,4	0,067
**Na biópsia renal**					
Proteinúria, g/24h	5,5 [3,1–6,5]	4,3 [2,1–9,6]	5,8 [4,2–6,7]	5,5 [2,5–7,6]	0,636
TFGe, mL/min/1,73 m^2^					0,051
< 30	4 (26,7)	8 (25,0)	11 (50)	23 (33,3)	
30–60	1 (6,7)	10 (31,3)	6 (27,3)	17 (24,6)	
> 60	10 (66,7)	14 (43,7)	5 (22,7)	29 (42,0)	
Valor absoluto da TFGe, mL/min/1,73 m^2^	106,9 [18,8–132,1]	50,9 [30,5–98,3]	33,6 [8,9–60,6]	54,1 [30,0–98,9]	**0,020**
Albumina sérica, g/dL	1,5 ± 0,7	2,2 ± 0,9	2,6 ± 0,9	2,2 ± 0,9	**0,001**
Hemoglobina, g/dL	12,0 ± 2,9	12,1 ± 1,8	10,6 ± 2,4	11,6 ± 2,3	**0,038**
HA	3 (23,1)	13 (38,2)	14 (63,6)	30 (43,5)	**0,029**

Abreviações – a: anos; me: meses; se: semanas; TFGe: taxa de filtração glomerular estimada; HA: hipertensão arterial. Notas – Valores para variáveis categóricas são apresentados como números (porcentagem); valores para variáveis contínuas como média ± desvio padrão, no caso de distribuição normal, ou mediana [intervalo interquartil], se distribuição não normal. Os valores de p foram obtidos pelo teste qui-quadrado de Pearson (ou correção pelo teste exato de Fisher) para variáveis categóricas e ANOVA unidirecional ou teste de Kruskal-Wallis para variáveis contínuas, conforme apropriado.

**Tabela 2:** Doenças subjacentes dos pacientes com amiloidose AA

	CA (1978–1991) (n = 15)	CB (1992–2005) (n = 32)	CC (2006–2019) (n = 22)	Total (n = 69)	p
**Doença subjacente**					
**Infecção crônica**	**7 (46,7)**	**14 (43,8)**	**12 (54,5)**	**33 (47,8)**	0,734
Tuberculose	4	6	4	14 (20,3)	
Bronquiectasia	1	4	3	8 (11,6)	
Abuso de drogas injetáveis	0	2	1	3 (4,3)	
Fibrose cística	0	0	2	2 (2,9)	
Úlceras crônicas	2	0	0	2 (2,9)	
Osteomielite	0	0	1	1 (1,4)	
Doença de Whipple	0	0	1	1 (1,4)	
Infecções pulmonares recorrentes	0	1	0	1 (1,4)	
Pielonefrite xantogranulomatosa	0	1	0	1 (1,4)	
**Doenças inflamatórias crônicas**	**4 (26,7)**	**17 (53,1)**	**7 (31,8)**	**28 (40,6)**	0,136
**Doenças reumáticas**	**4 (26,7)**	**13 (40,6)**	**4 (18,2)**	**21 (30,4)**	0,199
Artrite reumatoide	0	9	2	11 (15,9)	
Artrite idiopática juvenil	3	1	0	4 (5,8)	
Artrite psoriática	0	1	1	2 (2,9)	
Espondilite anquilosante	0	2	0	2 (2,9)	
Lúpus eritematoso sistêmico	1	0	0	1 (1,4)	
**Outras doenças inflamatórias crônicas**	**0 (0)**	**4 (12,5)**	**3 (13,6)**	**7 (10,1)**	0,336
Doença de Crohn	0	2	2	4 (5,8)	
Polimialgia reumática	0	0	1	1 (1,4)	
Hidradenite supurativa	0	0	0	1 (1,4)	
Paniculite associada à deficiência de α1-antitripsina	0	1	0	1 (1,4)	
Síndrome de Muckle-Wells (NLRP3 p.Arg262Leu)	0	1	0	1 (1,4)	
**Doenças malignas**	**2 (13,3)**	**0 (0)**	**2 (9,1)**	**4 (5,8)**	0,138
Carcinoma colorretal	1	0	0	1 (1,4)	
Câncer cervical	0	0	1	1 (1,4)	
Câncer de mama	0	0	1	1 (1,4)	
Linfoma não Hodgkin	1	0	0	1 (1,4)	
**Idiopáticas**	**2 (13,3)**	**1 (2,9)**	**1 (4,5)**	**4 (5,8)**	0,360

Notas – Os valores para variáveis categóricas são apresentados como números (porcentagens).

A duração mediana do acompanhamento foi de 37,7 meses (Tabela 3). Cerca de 74% dos pacientes evoluíram para DRET e 13% exibiram DRET na apresentação (Tabela 3). Apenas dois pacientes foram submetidos a transplante renal.

**Tabela 3:** Curso clínico e desfechos da amiloidose AA

	1978–1991 (n = 15)	1992–2005 (n = 32)	2006–2019 (n = 22)	Total (n = 69)	p
**Acompanhamento, me** ^a^	12,8 [4,0–229,2]	60,4 [8,3–151,1]	31,7 [11,2–74,7]	37,7 [8,2–134,7]	0,666
**Envolvimento extrarrenal**	4 (26,7)	9 (28,1)	14 (63,6)	27 (39,1)	**0,017**
Envolvimento de um órgão	3 (20,0)	4 (12,5)	11 (50)	18 (26,1)	**0,008**
Envolvimento de múltiplos órgãos	1 (6,7)	5 (15,6)	3 (13,6)	9 (13)	0,693
Cardíaco	0 (0)	6 (18,8)	5 (22,7)	11 (15,9)	0,286
Hepático	1 (6,7)	3 (9,4)	4 (18,2)	8 (11,6)	0,707
Neuropatia	2 (13,3)	0 (0)	1 (4,5)	3 (4,3)	**0,006**
Hipotensão ortostática	0 (0)	3 (9,4)	1 (4,5)	4 (5,8)	0,420
Trato gastrointestinal	1 (6,7)	3 (9,4)	6 (27,3)	10 (14,5)	0,145
Tireoide	–	3 (9,4)	1 (4,5)	4 (5,8)	NA^c^
**Desfechos clínicos**					
**Terapia renal substitutiva**					
DRET com necessidade de diálise	10 (66,7)	23 (71,9)	18 (81,8)	51 (73,9)	0,551
DRET na apresentação	1 (6,7)	3 (9,4)	5 (22,7)	9 (13,0)	0,255
DRET durante o acompanhamento	9 (60,0)	20 (62,5)	13 (59,1)	42 (60,9)	0,751
Transplante renal	1 (6,7)	1 (3,1)	0 (0)	2 (2,9)	0.157
**Óbito**	13 (86,7)	29 (90,6)	13 (59,1)	55 (79,7)	**0,014**
Infecção^d^	1 (9,1)	15 (48,4)	10 (76,9)	26 (47,3)	**0,002**
Insuficiência cardíaca	1 (9,1)	4 (12,9)	0 (0)	4 (7,3)	
Eventos isquêmicos	0 (0)	5 (16,1)	1 (7,7)	7 (10,1)	
Morte súbita ou arritmia cardíaca	2 (18,2)	0 (0)	1 (7,7)	3 (5,5)	
Insuficiência renal	1 (9,1)	1 (3,2)	0 (0)	2 (3,6)	
Diversos^e^	3 (27.3)	1 (3,2)	0 (0)	4 (7,3)	
Óbitos não relacionados à amiloidose	0 (0)	1 (3,2)	1 (7,7)	2 (3,6)	
Desconhecidos	5 (38,5)	2 (6,9)	0 (0)	7 (12,7)	

Abreviações – me: meses; DRET: doença renal em estágio terminal. Notas – Valores para variáveis categóricas são apresentados como números (porcentagem) e, para variáveis contínuas, como mediana [intervalo interquartil]. Os valores de p são obtidos pelo teste qui-quadrado de Pearson (ou correção pelo teste exato de Fisher) para variáveis categóricas e pelo teste de Kruskal-Wallis para variáveis contínuas, conforme apropriado. ^a^Fim do acompanhamento: agosto de 2020. ^b^Dados ausentes superiores a 25% (as porcentagens referem-se ao número total de pacientes). ^c^Não aplicável para esta categoria. ^d^Infecção incluiu sepse (n = 20), pneumonia (n = 4), empiema tuberculoso (n = 1) e peritonite (n = 1).^e^Causas diversas incluíram hemorragia pulmonar (n = 1), câncer colorretal (n = 2) e amiloidose (n = 1).

Conforme demonstrado na Tabela 4, o tempo mediano até a DRET foi de 31 meses. A sobrevida global mediana foi de 66 [IC 95% 33,0–99,0] meses, e a expectativa de vida desses pacientes foi de 64 [IC 95% 56,2–71,8] anos. As infecções foram a principal causa de óbito (Tabela 3).

**Tabela 4:** Dados de sobrevida dos pacientes com amiloidose AA

	CA (1978–1991) (n = 15)	CB (1992–2005) (n = 32)	CC (2006–2019) (n = 22)	Total (n = 69)	p
**Sobrevida global, me** ^a^	14 [IC 95% 0,0–131,4]	66 [IC 95% 48,0–84,0]	42 [IC 95% 0,0–104,2]	66 [IC 95% 33,0–99,0]	0,784
**Sobrevida renal, me** ^a^	103 [IC 95% 14,5–191,5]	22 [IC 95% 0,5–43,5]	26 [IC 95% 8,8–43,2]	31 [IC 95% 14,6 – 47,3]	0,504
**Sobrevida em diálise, me** ^b^	8 [IC 95% 0,0–18,8]	21 [IC 95% 0,5–41,3]	19 [IC 95% 8,6–29,4]	20 [IC 95% 12,0–28,0]	0,969
**Idade ao óbito, a** ^c^	52,3 [IC 95% 43,7–60,8]	59,0 [IC 95% 53,6–64,4]	61,4 [IC 95% 54,2–68,5]	58,3 [IC 95% 54,5–62,2]	0,217
Infecção crônica	58,3 [IC 95% 47,7–68,9]	62,4 [IC 95% 55,1–69,6]	56,4 [IC 95% 46,8–66,0]	59,4 [IC 95% 54,1–64,6]	0,727
Doenças inflamatórias crônicas	44,5 [IC 95% 29,7–59,3]	55,7 [IC 95% 47,6–63,8]	71,2 [IC 95% 62,2–80,2]	57,5 [IC 95% 51,1–63,9]	**0,041**
**Taxa de mortalidade em 1 ano/100 pacientes-ano**	8,97	12,5	11,4	11,2	
**Taxa de DRET em 1 ano/100 pacientes-ano**	10,98	17,4	24,7	17,2	

Abreviações – a: anos; me: meses. Notas – ^a^Valores são apresentados como média. ^b^Sobrevida em diálise de 51 pacientes com amiloidose AA, censurados no momento do transplante renal. ^c^Valores são apresentados como média.

### Comparação de Coortes

A incidência de amiloidose AA permaneceu estável na CA e na CB, em oposição à redução significativa na coorte mais recente (64%, 62,7% e 44%, respectivamente; p = 0,027), como mostrado na Figura 1.

**Figura 1:** Incidência de amiloidose AA nos últimos 40 anos.

### Características Basais e Doenças Subjacentes

Conforme mostrado na Tabela 1, a distribuição por sexo manteve-se estável nas três coortes, com homens e mulheres igualmente afetados. A idade média na apresentação aumentou de 40,9 anos na CA para 50,5 na CC (p = 0,095). Ao analisar os pacientes com base na causa subjacente, aqueles com doenças inflamatórias crônicas tiveram o aumento mais significativo na idade média de apresentação da doença renal, passando de 25,8 anos na CA para 54,5 anos na CC (p = 0,002).

A mediana da TFGe na biópsia diminuiu ao longo das três coortes, de 106,9 mL/min/1,73 m^2^ para 33,6 mL/min/1,73 m^2^ (p = 0,020) e, no momento da biópsia renal, mais pacientes da CC (50%) se encontravam no estágio 4 ou superior da DRC, comparados à CA (26,7%) (p = 0,051). A albumina sérica e a hemoglobina também apresentaram diferenças significativas entre as três coortes. A albumina sérica melhorou de uma média de 1,5 g/dL na CA para 2,6 g/dL na CC (p = 0,001), enquanto a hemoglobina diminuiu de 12,0 g/dL para 10,6 g/dL (p = 0,038). A hipertensão foi significativamente mais comum na CC (63,6%) do que na CA (23,1%) (p = 0,029).

Não houve alterações nas porcentagens relativas das doenças subjacentes entre as três coortes (Tabela 2). Infecções crônicas foram a principal doença subjacente, apresentando um leve aumento ao longo do tempo (de 46,7% na CA para 54,5% na CC), embora sem significância estatística. A incidência de tuberculose diminuiu da CA para a CB, observando-se que não houve casos decorrentes dessa infecção desde 2009.

### Curso Clínico e Desfechos

Todos os pacientes concluíram o estudo e não houve perdas de acompanhamento. O envolvimento extrarrenal foi mais frequente na CC (63,6%) do que na CA (23,1%) (p = 0,017) (Tabela 4). De modo geral, as infecções foram a principal causa de óbito, com um aumento significativo ao longo do tempo (9,1% na CA para 76,9% na CC, p = 0,002).

Não houve alterações estatisticamente significativas na sobrevida global e renal entre as três coortes (Tabela 4). A sobrevida renal mediana diminuiu de 103 para 26 meses, conforme o aumento na taxa de DRET em 1 ano/100 pacientes-ano. A sobrevida global mediana aumentou de 14 para 42 meses, e a taxa de mortalidade em 1 ano/100 pacientes-ano permaneceu estável, o que não foi estatisticamente significativo, mesmo considerando o envolvimento cardíaco. Os pacientes da CC foram a óbito em uma idade mais avançada (61,4 anos) do que os pacientes da CA (52,3 anos) (p = 0,217). Considerando a etiologia, a expectativa de vida média melhorou em pacientes com doenças inflamatórias crônicas subjacentes, de 44,5 anos na CA para 71,2 anos na CC (p = 0,041).

## Discussão

Nas últimas quatro décadas, a amiloidose AA foi o tipo mais frequente de nefropatia amiloide em nosso centro. Nossa incidência de biópsia de amiloidose renal AA foi de 56,1%, o que difere significativamente dos 7% relatados pela *Mayo Clinic* ^12^ e dos 12% relatados pelo *National Amyloidosis Centre* (NAC) do Reino Unido, embora a última série tenha incluído biópsias de tecido além do renal^13^. Observamos um declínio nos casos de amiloidose AA de 62,7% antes de 2006 para 44% após 2006. Isso se alinha às tendências contemporâneas relatadas pelo banco de dados do NAC do Reino Unido, embora permaneça excepcionalmente elevado^3^. No entanto, nossa taxa de incidência de amiloidose AA é de aproximadamente 1 caso por milhão de pessoas-ano, o que está de acordo com a incidência relatada da doença em países ocidentais^14^.

A série mais recente do NAC do Reino Unido relatou um declínio nos casos de amiloidose AA para 3% após 2016 e um aumento nos casos de amiloidose ATTRwt^3^. Em nossa série, a diminuição da incidência de tuberculose e o aumento de casos de amiloidose AL foram as principais causas da mudança epidemiológica nos últimos anos^9^. No entanto, a amiloidose AL permanece subdiagnosticada, considerando a incidência mínima estimada de 0,5 casos por 100.000 habitantes e nossa área de referência superior a 1 milhão de habitantes^15^. Os resultados de um recente painel Delphi aplicado a médicos portugueses de diferentes especialidades revelaram uma falta de consenso em relação ao diagnóstico e ao manejo precoce de pacientes com amiloidose AL, o que pode contribuir para seu subdiagnóstico^16^. Outra explicação para a maior incidência do tipo AA é a inclusão de pacientes com amiloidose comprovada por biópsia renal, o que pode superestimar a porcentagem relativa de amiloidose renal predominante como sendo do tipo AA. De modo geral, não temos um excesso de casos de amiloidose AA, mas sim um déficit no diagnóstico de outras formas de amiloidose com envolvimento renal, especialmente a AL.

Em nossa série, os pacientes eram significativamente mais velhos no momento do diagnóstico na coorte mais recente, com uma média de idade de 50,5 anos. Isso provavelmente reflete uma melhora no tratamento das condições subjacentes, principalmente doenças inflamatórias, e está em conformidade com outros relatos, embora nossos pacientes fossem mais jovens^11,14,17^.

Diferentemente de outros países desenvolvidos - onde predominam as condições inflamatórias - em nossa coorte, as doenças infecciosas permaneceram como a principal causa de amiloidose por AA nas três coortes^11,18^. Em termos gerais, a tuberculose foi a infecção mais comum, o que está relacionado ao período de estudo prolongado e à alta taxa de incidência de tuberculose em Portugal. A elevada frequência de amiloidose AA associada pode ter sido influenciada pela bronquiectasia pós-tuberculose, observada principalmente nas coortes mais antigas. No passado, a tuberculose foi documentada em muitas séries como a principal causa de amiloidose AA, mas essa incidência tem diminuído recentemente^5^. Em nossas instalações, não houve casos confirmados de amiloidose AA relacionada à tuberculose desde 2009.

Embora os casos de tuberculose tenham diminuído, novas infecções, como bronquiectasia e osteomielite crônica, tornaram-se mais prevalentes. Devido ao aumento da idade e das comorbidades, o controle da fonte nem sempre é possível, e a inflamação crônica persiste. Por outro lado, houve uma tendência de redução das condições reumatológicas crônicas - como a artrite reumatoide - como causas da forma AA, provavelmente atribuível à disponibilidade de DMARDs. Em contraste com as evidências crescentes, o abuso de drogas intravenosas não foi associado à amiloidose por AA em nossa coorte^11^.

Os pacientes diagnosticados mais recentemente eram mais velhos e apresentaram função renal reduzida na biópsia renal. Isso pode estar relacionado a um atraso no encaminhamento do paciente à nefrologia devido à idade mais avançada no momento da apresentação, comorbidades e baixa suspeita de amiloidose. A reserva funcional renal diminui com a idade, o que pode levar a uma perda mais rápida da função renal na presença dessa patologia^11^. A baixa hemoglobina e a prevalência de hipertensão na CC no momento da apresentação estavam provavelmente relacionadas à insuficiência renal mais avançada.

Em consonância com a idade mais avançada no diagnóstico, a idade ao óbito aumentou de 52,3 para 61,4 anos. Isso foi mais evidente quando a etiologia subjacente eram condições inflamatórias, refletindo um melhor controle da doença e complicações mais tardias, considerando o surgimento de novas terapias. No entanto, a sobrevida global não apresentou diferença significativa. Isso provavelmente esteve relacionado à idade mais avançada e à doença renal mais grave no momento da apresentação na última década. As mesmas explicações justificam nossos achados de sobrevida renal.

As doenças infecciosas foram a principal causa de óbito desses pacientes nos últimos trinta anos, com uma incidência crescente. A disponibilidade de DMARDs mudou significativamente o curso natural das doenças reumatológicas. O efeito imunossupressor cumulativo dos DMARDs, combinado com um aumento na expectativa de vida e na fragilidade relacionada à idade, pode ser uma explicação para essa tendência.

Reconhecemos certas limitações deste estudo realizado fora de um centro de referência para amiloidose hereditária^9^. O longo período do estudo e seu desenho retrospectivo são suscetíveis a vieses de informação, e os dados limitados disponíveis, particularmente de diagnósticos estabelecidos em décadas anteriores, restringiram a análise de algumas variáveis. Além disso, a seleção dos pacientes com base na biópsia renal restringiu o tamanho da amostra. No entanto, o principal ponto forte do estudo reside em sua natureza de longo prazo, com períodos de acompanhamento superiores aos de alguns dos estudos mais abrangentes sobre a sobrevida da amiloidose sistêmica^9^.

Em conclusão, os resultados de nossa coorte retrospectiva mostram uma diminuição na incidência de amiloidose AA no noroeste de Portugal. A expectativa de vida melhorou de modo geral, refletindo os avanços na terapêutica e no manejo desses pacientes. No entanto, o prognóstico renal permaneceu inalterado ao longo das décadas, sugerindo a necessidade de reconhecimento adequado, diagnóstico precoce e encaminhamento oportuno para a nefrologia, uma vez que a doença se manifesta em uma fase mais tardia da vida.
